# Transactions of the American Medical Association

**Published:** 1860-07

**Authors:** 


### Art. V.—*Transactions of the American Medical Association*, Vol. XII. Philadelphia, 1859, pp. 722.

The volume before us has one merit which was not characteristic of its predecessors—it is neither bulky nor overloaded with lengthy reports. All that the committee have deemed proper to publish occupies about three hundred pages less than were comprised in vol. XI. Without inquiring at this moment whether commendable judgment and discretion have been exhibited in the selection of materials for the present work, we may at least give credit to the committee for their consideration in supplying to the profession a publication which is neither unwieldly nor unnecessarily weighty. The usual component parts of all transactions, such as the minutes of the annual meeting, reports of officers, address of president, etc., occupy nearly one-fourth of the contents of the volume, while far more than half the book is occupied by "Observations on Malarial Fever," by Prof. Joseph Jones, of Georgia. But limited space has therefore been appropriated to other valuable papers, while some of the materials presented to the notice of the Association, and doubtless worthy of a place in the transactions, may have been omitted. It is true that a large number of the committees merely "reported progress," and were unable to present any subjects for the consideration of the Association, and this may account for the smaller dimensions of the volume for 1859. In this view, the lengthy dissection of the subject of malarial fever may have been a godsend to the committee, in their fears that the volume might not otherwise possess sufficient ponderousness.

The Association met in Louisville, on the 3d of May, 1859; the representation of delegates was therefore mainly Western in its character. It must always happen that the district of country in which the convention assembles will be most largely represented in its meetings, on account of the facilities of access. If this result has some disadvantages, it has also one advantage, that each portion of the Union is able at some one time or other to put forth its voice in behalf of local interests which might be overlooked, were some central spot chosen for the perpetual holding of the meetings. A local as well as a national character must thus necessarily, therefore, attach always to the American Medical Association, from its very constitution and regulations. The president, Dr. Miller, of Kentucky, in alluding to the assembling of so many "representatives of the great American medical profession, an army forty thousand strong," congratulated them that, although as a body they might not be so learned, so critically and nicely framed in all the minutiæ of the profession as their European *confrères*, yet, for strength, integrity, and precision in all the great principles guiding to a successful combat with disease, the body was equal if not superior to that of any kingdom of continental Europe. We might briefly sum up the proceedings of the Association, by alluding merely to those prominent points which give a personal identity to the twelfth annual meeting as distinctive from those which had preceded it; but at this time it is our purpose to speak only of the literary results which have flowed from it.

The address of Dr. Lindsly, the former president, refers to the rapid progress of medical knowledge and to the improved modes of treatment, etc., which the nineteenth century has called forth to relieve the pain of the suffering and to add to the comfort of the unfortunate. The report of the Special Committee on Government Meteorological Reports gives a historical sketch of the interest which the government has taken in scientific inquiries into meteorological phenomena, and of the co-operation of the Smithsonian Institute with the Army Medical Department and Patent Office, and suggests modes by which more accurate and reliable observations may be collected for comparison. The report on Criminal Abortion calls attention to the prevalence of a wide-spread system of destruction of fœtal life, and cites, as the main causes of this general demoralization, the popular belief, even among mothers themselves, that the fœtus is not alive till after the period of quickening, the supposed carelessness of the profession as to fœtal life and the omission of precautionary measures preventive of abortion, as well as the grave defects of all legal enactments in regard to the fœtus in utero. The subject is an important one, calling for the interposition of medical men everywhere through the country, with a view to the general suppression of this criminal interference with fœtal life.

California receives special attention as to its medical topography and epidemics from Dr. Thomas M. Logan, of Sacramento, in a report to which is attached considerable interest, "not only on account of the remarkable configuration of the surface of that State, and the varieties of climate met with within its area, but also because of the rapid transition stages, transpiring before our eyes, between the rudeness of the first settlements and the more refined condition of countries long subjected to civilization." Michigan receives a similar attention from Dr. J. H. Beech, of that State, in a carefully written report. The "Report on a Uniform Plan for Registration Reports of Births, Marriages, and Deaths," embraces all the points which have been deemed essential to a proper aggregation of facts bearing upon the vital statistics of the country, and supplies a plan for uniformity of details which has been long needed.

The remainder of the book—*four hundred and twenty pages*—is almost wholly devoted to the paper of Prof. Jones, to which we have previously alluded. Without wishing to detract for an instant from the valuable evidences of industrious research which are thus supplied to the profession, we do not think the committee have acted wisely in publishing it, after the Association had removed it from their consideration, as is exhibited in the "explanatory note by the Committee of Publication," in which they acknowledge that any jurisdiction they originally had over the paper had been taken away from them, and been bestowed upon the Committee on Prize Essays. The latter failed to make any report, and therefore the Committee on Publication "were compelled either to publish it as originally intended by the Association, or withhold it altogether." We contend that the latter alternative was the only one open to them, for they had no just reason—as a committee—to assume that anything they did, after the subject was taken out of their hands, was "more nearly in compliance with the expressed wish of the Association." We have spoken only of the action of those having the paper of Dr. Jones in charge. The merits of his investigations and analytical examinations of the important questions connected with malarial fever speak for themselves, while the amount of steady mental labor which must have been employed in analyzing the views of different writers upon the same subject, in comparing the results of their observations, and in the detailed description of new theories, can be readily imagined from a glance at the pages of his contribution. The paper, which deserves a careful perusal, is too long, however, for an extended notice, and the few brief remarks we could make upon it would fail to do justice to any merits it may possess.

